# Impact of the Ketogenic Diet on Neurological Diseases: A Review

**DOI:** 10.3390/life15010071

**Published:** 2025-01-09

**Authors:** Carmen Rubio, Alejandro López-Landa, Hector Romo-Parra, Moisés Rubio-Osornio

**Affiliations:** 1 Neurophysiology Department, Instituto Nacional de Neurología y Neurocirugía “Manuel Velasco Suárez”, Mexico City 14269, Mexico; mrubio@innn.edu.mx (C.R.); alexlanda10@hotmail.com (A.L.-L.); hector.romo.parra@gmail.com (H.R.-P.); 2 School of Medicine, Benemérita Universidad Autónoma de Puebla, Puebla City 72000, Mexico; 3 Psychology Department, Universidad Iberoamericana, Mexico City 01376, Mexico; 4 Neurochemistry Department, Instituto Nacional de Neurología y Neurocirugía “Manuel Velasco Suárez”, Mexico City 14269, Mexico

**Keywords:** ketogenic diet, neurological diseases, neuroglia, neuroinflammation

## Abstract

Background: The ketogenic diet (KD), high in fat and low in carbohydrates, was introduced in the 1920s as a non-pharmacological treatment for refractory epilepsy. Although its mechanism of action is not fully understood, beneficial effects have been observed in neurological diseases such as epilepsy, Alzheimer’s disease, and Parkinson’s disease. Objective: This review examines the impact of the ketogenic diet and its molecular and neuroglial effects as a complementary therapy for neurological diseases. Discussion: KD is associated with neuroprotective and antioxidant effects that improve mitochondrial function, regulate neurotransmitter flow, and reduce neuroinflammation and oxidative stress. Glial cells play an essential role in the utilization of ketone bodies (KBs) within the central nervous system’s metabolism, particularly during ketosis induced by the KD. Thus, the KD represents a broad and promising strategy that involves both neurons and glial cells, with a molecular impact on brain metabolism and neuroinflammatory homeostasis. Conclusion: Multiple molecular mechanisms have been identified to explain the benefits of the KD in neurological diseases; however, further experimental and clinical studies are needed to address various molecular pathways in order to achieve conclusive results.

## 1. Introduction

The insult inflicted on central nervous system (CNS) cells present in neurological diseases often leads to irreversible damage that is difficult to control with conventional treatments. This has necessitated the exploration of alternative, sophisticated treatments that can potentially mitigate damage as adjuvant therapies [1]. Over the past few decades, the ketogenic diet (KD) has emerged as a complementary therapy for a variety of systemic diseases, including metabolic disorders such as obesity [2] and diabetes [3], cardiovascular diseases [4], polycystic ovary syndrome [5], non-alcoholic fatty liver disease, and certain types of cancer [6,7,8]. Recently, benefits have also been reported for neurological diseases characterized by chronic neuroinflammation [9,10,11,12,13], such as epilepsy [14], Alzheimer’s disease (AD), Parkinson’s disease, traumatic brain injury, cerebral ischemia, multiple sclerosis (MS), Huntington’s disease (HD) [15], and brain tumors like gliomas [16,17]. Its effects as a complementary neuroprotective therapy in headaches and sleep disorders have yet to be fully established.

According to evidence, there is considerable variation and adaptation within the ketogenic diet [15], and no definitive classification has yet been established. However, the classic KD is characterized by a moderate protein percentage (6–15%), generally tailored to the patient’s requirements. Carbohydrates are drastically reduced (5–10%), making lipids the primary component of the diet (80–90%) [15]. These specific dietary requirements lead to changes in cellular energy metabolism, ultimately resulting in significant effects on inflammation and oxidation–reduction reactions [18]. During a KD, insulin levels decrease, and combined with an increase in adrenaline concentration, this leads to the release of free fatty acids (FFA), triacylglycerols (TAG), and glycerol from adipocytes. Once mobilized, these are used to produce ketone bodies (KBs), initially in the form of acetoacetate (AcAc), which is subsequently converted into beta-hydroxybutyrate and acetone [19].

The structure of ketone bodies, facilitated by monocarboxylate transporters (MCT-1) and sodium-dependent MCT-1 (SMCT-1), allows them to cross the blood–brain barrier, providing an alternative energy source for the brain [20]. Neuronally, a KD involves changes in the reconstruction of myelin sheaths and reduces inflammation by inhibiting pro-inflammatory cytokines [9,21] as well as certain molecular patterns. It enables alterations in excitatory and inhibitory neurotransmitter fluctuations [22], as well as the repair and formation of mitochondria, leading to a reduction in reactive oxygen species (ROS) [21]. Additionally, the caloric deficit resulting from a KD may have a neuroprotective potential by increasing the expression of neurotrophic factors [23].

A recent literature review highlighted the mechanisms through which the ketogenic diet can be used as an adjuvant therapy for certain neurological disorders [24]. Building on this as the frontier of current knowledge, the aim of this review is to explore the impact of cellular, molecular, and genetic effects (Table 1) on neurological diseases primarily observed in adulthood.

## 2. Ketogenic Diet in Epilepsy

The KD was first applied to epilepsy by Russell Wilder at the Mayo Clinic in 1921 [51], who proposed that ketones produced by the diet could be as effective as fasting in controlling epileptic seizures [52,53], as fasting had been suggested by Hippocrates as the only treatment to manage seizures [54,55,56]. Today, the ketogenic diet is used as a treatment option for drug-resistant epilepsy in children [57,58,59,60]. The benefits of the ketogenic diet for epilepsy are numerous [44], primarily attributed to the regulation of neurotransmitters and ion channels, enhancement of mitochondrial biogenesis, and reduction in oxidative stress, as well as the inhibition of apoptotic factors and genetic changes [25,61]. Recently, many benefits of the KD have been observed in neuroglia, suggesting that these modifications could lead to significant changes in epilepsy, which have not yet been fully addressed.

The ketogenic diet slows down energy availability, stabilizing and reducing neuronal excitability. The excitatory–inhibitory regulation of key neurotransmitters like glutamate and GABA allows the KD to enhance stability by modifying the seizure threshold [28]. The excitatory modifications primarily affect glutamate release by inhibiting the vesicular glutamate transporter 1 (VGLUT1) [44], leading to decreased release into the synaptic cleft. Beta-hydroxybutyrate directly inhibits voltage-dependent AMPA receptors [62], causing hyperpolarization and inhibition of excitatory neurotransmitter release [28]. Another glutamatergic effect involves the glial enzyme glutamine synthetase, which converts glutamate into glutamine; this glutamine is then transported to neurons and transaminated into alpha-ketoglutarate, further reducing glutamate concentration. Additionally, levels of serotonin, dopamine, and norepinephrine are also modified [63,64], as these neurotransmitters play an important role in neuronal excitability and contribute to seizure reduction.

In terms of inhibitory modifications, several GABAergic changes have been identified in patients on a ketogenic diet, including an increased rate of conversion from glutamate to GABA by glutamate decarboxylase [65], as well as elevated levels of GABA in the cerebrospinal fluid. Alterations in GABA transaminase activity have been found, which inhibit the degradation of GABA. The KD increases GABA levels in presynaptic vesicles, and upon release, activates GABAergic GABA_A_ receptors, thereby dampening convulsive activity. The KD appears to influence the NKCC1 transporter, which is upregulated in patients and animal models, impacting intracellular chloride concentration and GABAergic neurotransmission. The involvement of genes such as *SLC12A2*, which encodes *NKCC1*, and *GAD1* and *GAD2* for GABA synthesis, is crucial in this context. Additionally, ATP production has been observed, which ultimately acts on the adenosine-1 receptor (A1R) and affects neurotransmission. Experimental studies have shown that a ketogenic diet can increase agmatine levels in the hippocampus [65], which is considered an inhibitory neurotransmitter and has an anticonvulsant effect by inhibiting N-methyl-D-aspartate, histamine, and adrenaline receptors.

Another approach observed experimentally is an increase in mitochondrial metabolism, which enhances ATP production, activating ATP-sensitive potassium channels (K_ATP_) and leading to neuronal membrane hyperpolarization. The increase in K_ATP_ has been linked to the activation of inhibitory adenosine A1 receptors [33,66], modifications in the Bad protein (Bcl-2-associated death agonist) [37,67], as well as the Wnt/β-catenin signaling pathway [38], by enhancing glucose uptake in cortical neurons. This activation of K_ATP_ channels results in reduced cellular excitability through the efflux of positive ions [68]. The signaling of the Wnt pathway and K_ATP_ channel activity modulated by the KD involves genes such as *KCNJ11* and *CTNNB1* (β-catenin), which may contribute to reduced seizure activity and neuroprotective benefits in other disorders. The mTOR signaling pathway is also downregulated by the KD, as several studies have reported that KBs directly inhibit mTOR [39,42,69,70]. mTOR is well-known for its involvement in the pathogenesis of epilepsy [71], as its hyperactivation leads to focal cortical dysplasia, resulting in drug-resistant epilepsy [72]. Additionally, an increase in the expression of nuclear factor erythroid 2–related factor 2 (Nrf2) has been observed after a three-week KD treatment [61]. Nrf2, a transcription factor that induces the expression of a wide array of genes [33], regulates neuroinflammation by reducing oxidative stress and reactive oxygen species (ROS) [73,74].

Another effect of the ketogenic diet in epilepsy is the activation of K2P channels (two-pore domain potassium channels) [75], which enables a continuous flow of potassium ions across the cell membrane, establishing a hyperpolarized resting membrane potential [76,77]. The neuroprotection provided by the KD through modifications in mitochondrial activity allows for the inhibition of caspase-3 [78] and regulates the activation of the NLRP3 inflammasome [44] in neutrophils and macrophages, blocking its activation in response to PAMPs and DAMPs [79]. Finally, the KD increases fatty acid oxidation by raising polyunsaturated fatty acid (PUFA) levels, which can reduce neuronal excitability and mitigate seizures by blocking voltage-activated sodium and Ca++ channels, activating K2P channels, and enhancing Na+/K+ ATPase pump activity [40]. Experimental and human studies, whether in adults or children, have shown changes in gut microbiota following a KD, although bacterial changes varied by study [48,80,81,82]. Further studies are needed to clarify the relationship between gut microbiota and its anticonvulsant effects.

At the clinical level, controlled clinical trials have focused on pediatric patients with drug-resistant epilepsy (DRE) [83,84,85,86,87]. Most studies report a high dropout rate [83], primarily due to the challenges of adhering to such a strict diet and the adverse symptoms that may arise, including constipation, gastroesophageal reflux, hypercholesterolemia, lack of energy, and vomiting [83,85]. However, patients who completed the studies demonstrated significant benefits, with seizure reductions exceeding 50% [83]. Systematic reviews with meta-analyses have provided a rigorous perspective on this approach [88,89,90,91,92]. While numerous reviews present substantial evidence, some fail to yield conclusive results [89]. Nevertheless, the majority highlight the positive efficacy and safety of the diet in epilepsy, citing reductions in seizure frequency, as well as improvements in cognition and behavior [88,91,93]. The primary challenge remains poor adherence [94] and a decline in quality of life due to gastrointestinal side effects, weight loss, and metabolic disturbances [88]. Many of these issues, however, can be managed, allowing patients capable of adhering to the diet to achieve its beneficial outcomes [95,96,97].

## 3. Ketogenic Diet in Alzheimer’s Disease

Alzheimer’s disease (AD) is associated with uncertain pathophysiological mechanisms. The deposition of amyloid-beta (Aβ) peptides and tau protein [98], inflammation, mitochondrial dysfunction [99,100], and glucose metabolic deficiency [101] represent key targets for therapeutic management through the ketogenic diet. AD leads to glucose hypometabolism, resulting in reduced glucose uptake and insulin resistance [102], deficiencies in glycolysis, and impairment of the GLUT1 glucose transporter [103], ultimately manifesting as deficits in higher cognitive processes. The KD compensates for glucose deficiency by providing an alternative energy source through ketone bodies [11,104], which mitigates the damage caused by this deficiency. Experimental studies [105,106] have shown a reduction in Aβ volumes in brain homogenates from murine models [9], as well as decreased levels of Aβ and tau in the brains of rodents treated with a ketogenic diet, accompanied by improvements in cognitive function after as little as 40 days of treatment [29]. This reduction in the accumulation of hallmark structures results in the blockade of neurotoxicity, thereby preventing synaptic dysfunction and neuronal loss [13,99].

On the other hand, an increase in angiogenesis and capillary density has been observed [107]. The M2 phenotype of microglia can secrete IL-10 and proangiogenic factors, such as VEGF, to suppress excess response or repair damaged tissue, in this case, the deposition of proteins [34]. Therefore, an increase in angiogenesis may enhance the quality of higher cognitive processes. In neurodegenerative diseases like Alzheimer’s, the KD could influence glial genes such as *SOD2*, which is involved in antioxidant defense, and genes related to mitochondrial function and stress response, such as PINK1 and DJ-1. This could reduce oxidative damage and promote neuronal survival. Additionally, astrocytes metabolize KBs to provide energy to neurons, highlighting the role of genes like GLUT1 and MCT1 in the transport of glucose and ketone bodies. In Alzheimer’s, the KD could regulate inflammatory genes in microglia, such as IL-1β and TNF-α, helping to reduce neuroinflammation.

Beta-hydroxybutyrate binds to the hydroxycarboxylic acid receptor 2 (HCA2) on microglial cells, and its activation reduces inflammatory activity. It has the ability to inhibit NLRP3, thus preventing the formation of the inflammasome protein complex and blocking the conversion of pro-IL-1β and pro-IL-1 into IL-1β and IL-18. At the nuclear level, beta-hydroxybutyrate activates the peroxisome proliferator-activated receptor gamma (PPARγ), which inhibits the nuclear expression of NFκB. This transcription factor controls several genes involved in inflammation, which may be related to the pathogenesis of Alzheimer’s disease (AD), so its negative regulation is another beneficial effect of the ketogenic diet on AD through PPARγ [41]. The neuroprotective effect of beta-hydroxybutyrate occurs through the increased gene transcription of NT-3, BDNF, and GDNF. The genetic effects are likely due to the activation of Nrf2, which in turn stimulates endogenous antioxidant systems such as glutathione, thioredoxin, peroxiredoxin, and heme oxygenase-1 [47], as well as the regulation of gene expression involved in oxidative stress and ROS. These downstream modifications in cellular signaling and genetic pathways by Nrf2 likely lead to alterations in the APOE2 gene, the most commonly associated gene with the apolipoprotein linked to the risk of earlier onset of the disease [108], as well as in the genes coding for *APP, Presenilin 1* (*PSEN1*), and *Presenilin 2* (*PSEN2*), which lead to excessive production of Aβ [49], mitigating complications and poor prognosis associated with AD. Similarly, the increasing expression of uncoupling proteins (UCP) leads to the transcription of many genes related to oxidative metabolism and the negative regulation of ROS production by reducing the mitochondrial membrane potential [13].

## 4. Ketogenic Diet in Parkinson’s Disease

**Figure 1:**
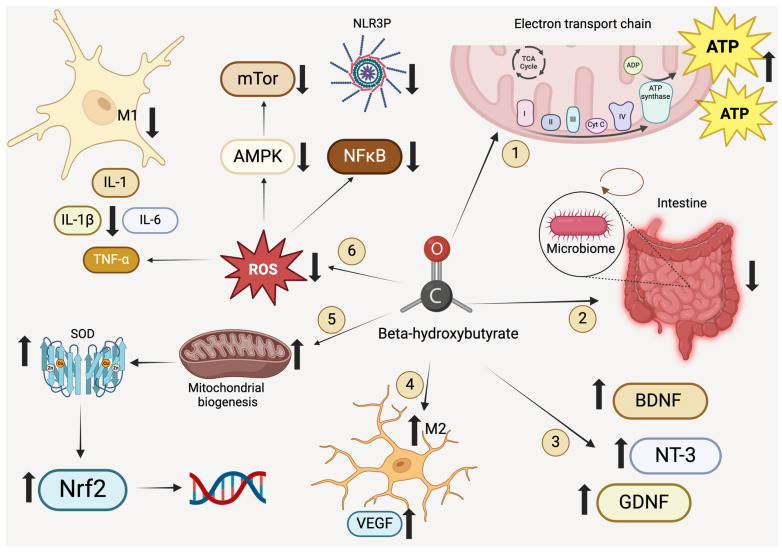
Effects of the ketogenic diet on neuroinflammation and oxidative stress. 1. The ketogenic diet bypasses the mechanistic failure of the electron transport chain, thereby providing an alternative energy source (ATP) through ketone bodies. 2. It reduces intestinal inflammation and modifies the gut microbiota. 3. Ketone bodies promote an increase in BDNF, GDNF, and NT-3, leading to a reduction in demyelination and axonal degeneration. 4. There is suppression of the M1 microglial phenotype, while the M2 phenotype is enhanced and activated, reducing and inhibiting inflammation and promoting angiogenesis through VEGF expression. 5. It increases mitochondrial biogenesis, enhancing superoxide dismutase (SOD) levels and activating anti-inflammatory pathways such as Nrf2 and its respective signal transduction and gene expression. 6. It decreases reactive oxygen species (ROS), inhibiting inflammatory cytokines such as IL-1, IL-6, IL-1β, and TNFα. Additionally, it can activate MAPK pathways, inhibit the mTOR pathway, and suppress the NF-κB pathway and the NLRP3 inflammasome. Created with https://www.biorender.com/.

Parkinson’s disease (PD) has a multifactorial pathophysiology involving multiple molecular mechanisms that result in both motor and non-motor symptoms [109]. These mechanisms include degeneration of nigrostriatal dopaminergic neurons, aggregation of α-synuclein protein [110,111,112], reactive microglia with neuroinflammation [113], oxidative stress, mitochondrial dysfunction, synaptic issues, brain energy deficits, and intestinal dysbiosis [114]. The ketogenic diet counteracts these mechanisms by providing energy compensation and bypassing Complex I activity failure [26] (Figure 1). Ketone bodies reduce the production of free radicals by improving mitochondrial respiratory efficiency, enhancing the oxidation of nicotinamide adenine dinucleotide (NADH) [30], and exerting antioxidant effects through the increased activity of glutathione and glutathione peroxidase. Additionally, the KD boosts neurotrophins like BDNF, NT-3, and GDNF, as well as proteins that prevent the aggregation of potentially toxic polypeptides (chaperones). The ketogenic diet can modify cellular signaling pathways, such as the mTOR pathway, involved in autophagy and mitochondrial renewal through mitophagy. It promotes compromised activity of pro-apoptotic factors and the prevention of inflammatory mediators like interleukins and tumor necrosis factor alpha. Finally, the KD may have potential and indirect roles in neurotransmission by reducing GABAergic activity from striatal neurons to the internal segment of the globus pallidus (GPi) and the pars reticulata of the substantia nigra (SNpr).

The ketogenic diet has been used as a complementary therapy for this neurodegenerative disease to improve both motor and cognitive symptoms. However, evidence from experimental research has shown that beta-hydroxybutyrate acts in vitro as a neuroprotective agent against the toxicity of 1-methyl-4-phenyl-1,2,3,6-tetrahydropyridine (MPTP) in dopaminergic neurons [115]. In one study [116] on mice with MPTP-induced toxicity, the administration of beta-hydroxybutyrate reduced neurotoxicity by improving cellular respiration and ATP production, along with enhanced motor skills and increased dopamine volume in the midbrain. In a rat model of Parkinson’s disease, a beneficial effect of the ketogenic diet on motor function was observed [26], as well as neuroprotection against the toxic effects of 6-hydroxydopamine through the activity of glutathione [32]. In a study involving lipopolysaccharide (LPS)-induced rats treated with beta-hydroxybutyrate, motor dysfunction was significantly improved by inhibiting microglial activation [117]. Another study [50] on an MPTP-induced mouse model of PD treated with the ketogenic diet found protection from motor dysfunction, dopaminergic neuronal loss, inflammation, and the reversal of dysbiosis, demonstrating a neuroprotective role through the diet-gut–brain axis [50].

Evidence from five clinical studies has shown that the ketogenic diet provides significant benefits, demonstrating a notable relationship between the reduction in the severity of Parkinson’s disease and a low-carbohydrate, high-fat diet [10,112,118,119,120]. Significant improvements were observed in the MDS-UPDRS scores for Parts I, II, III, and IV [10], as well as in cognitive performance, with a significant reduction in body weight [112] and improved voice quality, as measured by the Voice Handicap Index-10 [119]. Another study found some improvement in the Unified Parkinson’s Disease Rating Scale (UPDRS) scores [120]. However, contradictory results were observed in motor symptoms (MDS-UPDRS Part III) [10,112]. Two studies showed positive trends in reducing general PD symptoms, improving chronic disease biomarkers, and alleviating anxiety [121]. Additionally, improvements in cognition, mood, motor and non-motor symptoms, pain, anxiety, and overall quality of life were noted [122]. Recent systematic reviews [123,124] on the ketogenic diet in Parkinson’s disease showed a significant improvement in motor function, whether through vocal quality, gait, freezing, tremor, and/or balance, as well as improvements in non-motor symptoms (fatigue, cognition, urinary problems, pain, and daytime sleepiness). However, these reviews indicate that the wear and tear from the strict adherence to the diet, along with the sample size and duration, make it unreliable to generalize these findings to all PD patients [123].

The evidence is clear, and despite some contradictory results, the benefits of the ketogenic diet in Parkinson’s disease (PD) have been established. However, more studies are needed to explore other potential effects, which, though theoretical so far, are likely implicated and have not yet been thoroughly investigated, such as the effects of neuroglia through the KD in PD. Recently [113], an experimental study in rats with an LPS-induced PD model showed that the KD suppressed the inflammatory response and exerted neuroprotective effects by modulating the Akt/GSK-3β/CREB signaling pathway mediated by histone acetylation of the mGluR5 gene promoter region. The ablation of mGluR5 could stimulate microglial activation [125], and the results suggested anti-inflammatory effects of the ketogenic diet with upregulation of the mGluR5/Akt/GSK-3β/CREB signaling pathway by increasing mGluR5 gene acetylation while inhibiting microglia [113]. In another study [126] with early PD animal models induced by 6-OHDA injection into the medial forebrain bundle, a KD was administered for 3 weeks before and 4 weeks after the surgery. The study found that the ketogenic diet did not protect against 6-OHDA-induced dopaminergic neuron damage but tended to improve locomotor activity and normalize DA turnover in the striatum. Therefore, the ketogenic diet could potentially be therapeutically useful in supporting late-stage compensatory mechanisms via glial cells, but it does not necessarily act directly against oxidative stress-induced parkinsonian neurodegeneration [126].

## 5. Ketogenic Diet in Multiple Sclerosis

Multiple sclerosis (MS) involves an autoimmune response and neuroinflammation, leading to demyelination, edema, and axonal injury [127], with associated damage to the blood–brain barrier (BBB) [128]. Metabolic conditions and environmental factors should not be overlooked [129], as recent findings suggest that lifestyle can influence pathological inflammatory mechanisms and hormonal changes, improving the clinical expression of the disease [130,131]. The ketogenic diet has been shown to modulate certain inflammatory regulators, such as NF-κB inhibitor alpha (Nfkbia), tissue inhibitor of metalloproteinases-3 (TIMP-3), TNF-α, IL-6, COX-2, iNOS, VCAM-1, and ICAM-1 [132,133]. Additionally, its anti-inflammatory effects have been attributed to adenosine [134]. Adenosine has been demonstrated to reduce systemic inflammation by modulating LPS-induced transmigration of polymorphonuclear cells and decreasing pro-inflammatory mediators, such as TNF-α, IL-6, and CXCL2/3 [135]. Beta-hydroxybutyrate enhances mitochondrial respiration and NF-κB activation through the stimulation of the histone acetyltransferase p300/EP300, which subsequently promotes the synthesis of brain-derived neurotrophic factor (BDNF) [136]. BDNF is critical for synaptic plasticity, neuronal function, neurodevelopment [137,138], glucose homeostasis, energy balance [139], and neuroprotection by preserving myelin integrity [140,141] and neuronal regeneration [142]. Notably, some authors have highlighted the role of BDNF in promoting the remyelination of MS-induced lesions [142,143].

In experimental models, the ketogenic diet has shown beneficial effects. These analyses were conducted using experimental autoimmune encephalomyelitis (EAE) models and cuprizone (CPZ) mouse models. Both models involve demyelination; however, this represents a limitation as they do not fully replicate the pathophysiological characteristics of the disease. In studies using EAE models [46,144,145], findings demonstrated that the ketogenic diet protected against motor and vision loss by reducing immune cell infiltration and preserving myelination in the optic nerve [144]. Additionally, improved motor performance was observed compared to mice on a standard diet. Motor function was monitored daily using a motor disability scale and swimming speed measurements. It was noted that EAE mice on a KD showed recovery of swimming speed 17 days post-induction [145]. Another study evaluated neuronal energy metabolism, revealing symptom mitigation, increased immune cell populations (CD4+, CD8+, and CD45+) in the spinal cord, and a reduction in average lesion size in EAE mice [46].

Studies using the CPZ model with the ketogenic diet found that mice treated with a KD alongside CPZ demonstrated improved learning, memory, and motor performance, as well as reduced anxiety compared to those treated with CPZ alone [146,147]. These studies reported reduced activation of microglia and reactive astrocytes, increased expression of mature oligodendrocytes, and decreased apoptosis in oligodendrocytes. These effects were associated with downregulation of TRPA1 and PARP expression, inhibition of the p38-MAPK/JNK/JUN pathway, and activation of PI3K/AKT/mTOR signaling [146]. Another study found reduced anxiety, significant improvement in motor coordination, and reversal of learning and memory deficits with the KD [147].

A substantial body of clinical studies on the KD and MS has been published, ranging from case reports [148] to controlled clinical trials [149]. One case report described improvements in motor function and Expanded Disability Status Scale (EDSS) scores [148]. In controlled clinical trials, a KD intervention showed no new or enlarging lesions on magnetic resonance imaging (MRI), improvements in fatigue and depression scores, and significant enhancement in EDSS scores, specifically due to better bowel/bladder and sensory functions [149]. These authors extended their research in a Phase II trial [150], evaluating the KD’s effects on symptoms and leptin levels in 64 patients with relapsing-remitting MS. The results demonstrated significant reductions in body mass index (BMI), waist circumference, fat mass, and resting metabolic rate following the intervention. Additionally, there was a nearly 50% reduction in fatigue, and quality-of-life scores for physical and mental health significantly improved. EDSS scores showed reductions as well. Serum levels of leptin, insulin, hemoglobin A1c, and fasting triglycerides significantly decreased in patients following the diet [150].

Other studies have reported clinically significant improvements in the health-related quality of life questionnaire (MS-54) following the ketogenic diet [151,152]. A lower prevalence of poor sleep quality and daytime sleepiness, as well as enhanced psychological well-being and overall quality of life, has also been documented [153,154]. In one study comparing groups on different diets, the ketogenic diet group successfully maintained nutritional ketosis throughout the study. They exhibited lower blood glucose concentrations at weeks 4 and 12 compared to baseline, along with reduced plasma insulin levels at the same time points [35]. Another study [35] analyzed the microbiome in stool samples compared with healthy controls. Among those following the KD, the concentrations of nearly all fecal bacterial phyla analyzed decreased but began to increase as early as 12 weeks after starting the KD. A separate study [155] reported both the benefits and side effects of the ketogenic diet. Of participants, 47% noted improvements in MS-related symptoms, including paresthesia/pain, numbness, balance, headache frequency or severity, urinary urgency, muscle spasms, and vision. Furthermore, 62.5% reported weight loss, increased energy, and improved sleep quality while on the diet [155].

Although the experimental and clinical evidence is fairly redundant, it has proven more conclusive than in other neurological diseases, despite certain limitations. These include small population sizes, the challenges associated with adherence to the diet due to its multiple complications, and the absence of an exact model for MS in experimental studies. This highlights the need for further evidence to fully elucidate the mechanisms underlying the KD’s effects. Recently [156], elevated glutamate levels have been linked to regions of greater demyelination and axonal degeneration in MS [157]. This is attributed to increased production of IL-6 and IL-17A, which reduce glutamate uptake by astrocytes and stimulate its release at extrasynaptic sites [31,158]. On the other hand, the KD has been shown to increase BDNF levels, promoting the proliferation, migration, and differentiation of oligodendrocyte precursors at sites of myelin damage [43]. In an EAE model study [27], the KD was found to shift microglial polarization toward the protective M2 phenotype and to modulate the inflammatory environment by downregulating the production of pro-inflammatory cytokines, including TNF-α, IL-1β, and IL-6, while upregulating the release of anti-inflammatory cytokines such as TGF-β [27]. If the observed improvements with the ketogenic diet are linked to neurotransmitter regulation and microglial activity, the benefits achieved in glial cells and astrocytes should be considered. These cellular changes play a crucial role in driving meaningful improvements in the progression of neurological diseases.

## 6. Ketogenic Diet in Huntington’s Disease

Huntington’s disease (HD) is distinguished from other neurological diseases by the degeneration of medium-sized spiny striatal neurons [159], attributed to a genetic origin due to a mutation in the huntingtin gene (HTT) on chromosome 4p, leading to a polyglutamine expansion in the HTT protein [160]. Additionally, degeneration of striatal cells has been associated with impaired mitochondrial function, increased oxidative stress, heightened neuronal excitotoxicity [161], and disruption of the electron transport chain in the caudate and putamen [162]. Recently, it has been found that patients and experimental models with this disease may present a metabolic imbalance [163,164,165], including deficits in insulin secretion [166], loss of the ability to balance free glucose with stored glycogen [167], and alterations in the cholesterol biosynthetic pathway [168]. Since cholesterol is essential for maintaining the integrity of neuronal cell membranes and is involved in myelin formation, neurosteroid production, and synaptic activity [169], dietary changes that may influence these factors, such as the ketogenic diet and others with demonstrated neuroprotective effects, have been explored. As previously described, this diet has the potential to address the complementary pathological needs of this disease due to its effects on oxidative stress, neuroinflammation, and mitochondrial dysfunction.

A study examined sleep/wake cycle changes in a BACHD mouse model of HD, fed with either a standard diet or a ketogenic diet for 3 months [36]. The findings showed an increase in serum β-hydroxybutyrate levels and changes in the mice’s microbiome. The KD increased daytime sleep and improved sleep onset timing through the expression of the sleep-regulated gene (*Homer1*). Additionally, the KD improved activity rhythms, including rhythmic strength, and reduced inappropriate daytime activity and onset variability. The KD also enhanced motor performance in the rotating and challenging beam tests and had a significant impact on genes involved in various physiological responses, including inflammation and stress (Il6ra, Nos1, and Npy) [36]. Another study [170] of in vitro and in vivo experiments examined lipid metabolism (cholesterol pathway) in an R6/2 transgenic mouse model of HD. These mice were supplemented with cholesterol and beta-hydroxybutyrate to assess the chronic effects of a ketogenic diet over an 11-week period. The study found a delay in the onset of certain peripheral HD symptoms, including weight loss and some behavioral indices [170]. In an experimental study with an R6/2 1J transgenic mouse model of HD [119], where mice were fed a ketogenic diet ad libitum starting at six weeks of age, the KD improved working memory deficits, delayed weight loss, and did not cause any negative effects on behavioral parameters such as locomotor activity and coordination.

To date, there is only one case study of a patient with HD treated with a ketogenic diet, which showed a 52% improvement in motor symptoms according to the Total Motor Score on the Unified Huntington’s Disease Rating Scale (UHDRS) [171], as well as improvements in activities of daily living, HD-related behavioral problems, and mood-related quality of life. Although these results are promising, more controlled clinical studies are needed for the ketogenic diet to be considered conclusive for this disease. In addition, various HD mouse models have shown progressive alterations in glutamatergic transmission along the corticostriatal pathway [113,114], as well as changes in GABAergic synaptic activity. The increased inhibition dampens striatal output and may explain some of the behavioral symptoms [115,116]. Therefore, reducing intrastriatal GABA transmission may be a potential therapeutic target. Possibly, due to the changes and regulation of neurotransmitters facilitated by the ketogenic diet through astrocytes and other behavioral phenomena, this diet may offer regulatory effects for this disease, although these mechanisms remain to be fully explored.

## 7. Discussion

**Figure 2:**
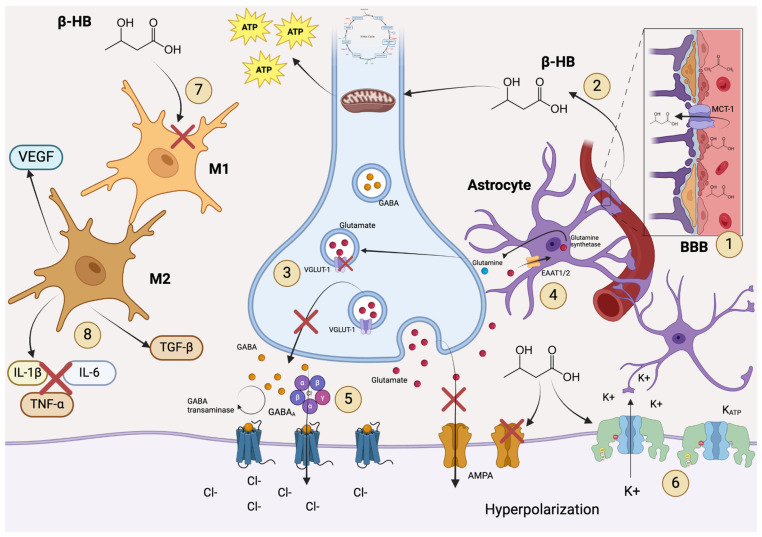
Effects of the ketogenic diet on neurological diseases. 1. Ketone bodies cross the blood–brain barrier through monocarboxylate transporters. 2. Beta-hydroxybutyrate and acetate enter the mitochondria and, through the Krebs cycle, facilitate the production of NADH and consequently ATP via the electron transport chain. This alternative energy production compensates for the energy deficiency present in neurological diseases. 3. It enables excitatory–inhibitory regulation by modulating neurotransmitters, primarily by regulating glutamate through the inhibition of vesicular glutamate transporter 1 and AMPA receptor activity. 4. Astrocytes participate in the glutamate-glutamine cycle by recapturing glutamate released into the postsynaptic space and converting it into glutamine via glutamine synthetase, which is then released and recaptured by presynaptic terminals. 5. It increases the expression of GABA_A_ receptors and enhances their uptake through glutamine transaminase, thereby allowing greater Cl^−^ influx and inducing hyperpolarization. 6. There is a marked increase in ATP-sensitive Potassium channels, further raising the seizure threshold. 7. Ketone bodies reduce type 1 microglial activation. 8. There is a significant increase in the expression of type 2 microglia, leading to decreased expression of pro-inflammatory cytokines such as IL-1β, IL-6, and TNFα, as well as anti-inflammatory cytokines like TGF-β and pro-angiogenic factors such as VEGF. Blood–brain barrier (BBB), acetate (AcAc), Nicotinamide Adenine Dinucleotide Reduced (NADH), Adenosine Triphosphate (ATP), vesicular glutamate transporter 1 (VGLUT1), α-amino-3-hydroxy-5-methyl-4-isoxazolepropionic acid (AMPA), Gamma-Aminobutyric Acid Type A (GABA_A_), ATP-sensitive Potassium Channels (K_ATP_), type 1 microglia (M1), type 2 microglia (M2), Interleukin 1 beta (IL-1β), Interleukin 6 (IL-6), Tumor Necrosis Factor alpha (TNFα), Transforming Growth Factor beta (TGF-β), Vascular Endothelial Growth Factor (VEGF). Created with https://www.biorender.com/.

Currently, the benefits of the ketogenic diet (KD) on the pathophysiology of neurological diseases are presented and expressed through a large number of experimental and clinical studies, many of which provide significant findings; however, they also have factors that prevent the global results from being conclusive. The pathophysiological mechanisms of neurological diseases are vast, and it is likely that the role of neuroglial cells is being overlooked in the search for yet-to-be-found answers (Figure 2). The fact that glial cells make up almost 50% of the total brain volume [172] is being omitted. Regardless of their support to the nervous system, glial cells contribute to homeostasis by controlling neurotransmitter flow, removing or repairing damaged neurons, interacting in neuronal signaling processes, modulating synaptic activity, providing cellular defense and repair, and regulating neuroinflammation.

Astrocytes are the most abundant glial cells in the brain, and their regulation of neurotransmitters gives them the ability to improve conditions like epilepsy and Parkinson’s disease through the modulation of the ketogenic diet. The supply of substrates to neurons for oxidative phosphorylation and the regulation of blood flow to meet the neuronal energy demand [173] enable the regulation of oxidative stress and ROS in neurological diseases. It has been discovered that astrocytes are the main site of fatty acid oxidation in the central nervous system, so the metabolic effect of the ketogenic diet in the brain takes place primarily within this glial population. Through ketosis, regulation of the astrocytic glutamate-glutamine cycle, glutamine synthetase activity, and the function of the vesicular glutamate transporter [174] occurs, altering the seizure threshold and membrane excitability.

Microglial reactivity and the increase in neuroinflammation are involved in multiple neurological diseases [126]. Its regulation by the ketogenic diet has been reported to exert anti-inflammatory effects; the KD may suppress M1 microglial activation, subsequently promoting M2 activation. This regulation of the M1 to M2 phenotype transition could mitigate subsequent neuroinflammatory changes and neuronal death [175]. It has been demonstrated that beta-hydroxybutyrate inhibits histone deacetylation and activates receptors such as GRP109A (HCA 2) [176]. This leads to consequences in cellular signaling pathways, such as NF-kB, and a decrease in the production of pro-inflammatory cytokines and pro-apoptotic substances. This results in an improvement in immune function and maturation, serving as a neuroprotective factor in different microglial phenotypes [177,178].

So far, the extensive body of experimental studies has highlighted numerous theories and proposals regarding the molecular and cellular mechanisms through which the ketogenic diet exerts its effects, ultimately leading to a reduction in the complex pathophysiological processes underlying neurological diseases [24]. Clinically, many of these theories have not been fully validated due to controversies in the outcomes of controlled clinical trials, as well as biases arising from limited sample sizes and insufficient exposure times to the diet. Poor adherence and compatibility of this dietary approach with patients’ lifestyles pose significant challenges when interpreting findings. The monotony of the diet, the social difficulties of adhering strictly to its requirements, short- and long-term side effects, and the lack of perceived benefits can negatively impact quality of life by increasing stress and anxiety associated with such a restrictive regimen. Consequently, adherence to this strict diet represents a significant challenge.

Further experimental studies exploring distinct molecular mechanisms are needed to achieve more conclusive results. Additionally, more robust controlled clinical trials with larger sample sizes and longer durations are essential. Providing patients with therapeutic, professional, and educational support, mitigating or managing side effects, and offering a more diverse and creative selection of permissible foods could enhance adherence to the diet, ultimately leading to higher-quality and more definitive outcomes.

## 8. Conclusions

Throughout the reviewed studies, it has been demonstrated that the ketogenic diet (KD) can positively influence the pathophysiology of disorders such as epilepsy, Alzheimer’s, Parkinson’s, and others. In particular, ketone bodies, such as beta-hydroxybutyrate, play a key role in reducing oxidative stress, modulating neuronal excitability, improving mitochondrial function, and promoting neuroprotective processes. Additionally, the ketogenic diet directly influences neuroglia, especially astrocytes and microglia, cells crucial for maintaining brain homeostasis. However, despite advancements in understanding the ketogenic diet in neurological diseases, there are still gaps in knowledge, particularly regarding the specific molecular mechanisms involved in the observed effects. The variability in study results and methodological limitations underscore the need for more experimental and clinical research to better understand the effects of the KD on the neurobiology of these disorders. Anti-inflammatory therapy, particularly through the modulation of neuroglia, appears to be a key area for future neuroprotective therapeutic strategies.

## Figures and Tables

**Table 1:** Comparative effects of the ketogenic diet in epilepsy, Alzheimer’s disease, Parkinson’s disease, multiple sclerosis and Huntington’s disease.

	Epilepsy	Alzheimer’s Disease (AD)	Parkinson’s Disease (PD)	Multiple Sclerosis (MS)	Huntington’s Disease (HD)
Mechanism of action	Slows down energy availability, stabilizes, and reduces neuronal excitability [25].	Compensates for glucose deficiency by providing alternative energy with KBs [11].	Eluding energy failure and Complex I activity [26].	Modulates inflammatory regulators such as Nfkbia, TNFα, IL-6, COX-2, iNOS, VCAM-1, and ICAM-1 [27].	Compensates for glucose deficiency by providing alternative energy with KBs [11].
	Excitatory–inhibitory regulation of key neurotransmitters such as glutamate and GABA [28].	Decreased levels of Aβ and Tau [29].	Reducing free radicals by improving mitochondrial respiratory efficiency by increasing NADH [30].	Anti-inflammatory effects by adenosine reducing systemic inflammation by modulating LPS and reducing CXCL2/3 [31].	Antioxidant effects due to increased glutathione and glutathione peroxidase activity [32].
	Increase in ATP, activating KATP, leading to hyperpolarization of the neuronal membrane [33].	Increased angiogenesis and capillary density by the M2 phenotype of microglia secreting IL-10 and VEGF [34].	Antioxidant effects due to increased glutathione and glutathione peroxidase activity [32].	Beta-hydroxybutyrate induces NF-κβ upregulation, activating histone acetyltransferase p300/EP300 and BDNF synthesis [35].	Regulation of inflammation and stress genes such as *Il6ra*, *Nos1*, and *Npy* [36].
	Modifications of the Bad protein, Wnt/β-catenin signaling pathway, mTOR, and Nrf2 [37,38,39].	Influence on antioxidant defense and mitochondrial function genes such as SOD2, PINK1, and DJ-1 [34].	Increase in neurotrophins such as BDNF, NT-3, GDNF, and chaperones [23].	BDNF enables synaptic plasticity, neuronal function, neurodevelopment, glucose homeostasis, and neuroprotection through myelin integrity [31].	Expression of the sleep-regulated gene *Homer1* [36].
	Activates K2P channels, leading to a continuous flow of potassium ions across the cell membrane, causing hyperpolarization [40].	Regulation of inflammatory genes in microglia, such as IL-1β and TNF-α, helping to reduce neuroinflammation [41].	Modify cell signaling pathways, such as the mTOR pathway [42].	BDNF upregulation allows remyelination of MS-induced lesions [43].	Increase in neurotrophins such as BDNF, NT-3, GDNF, and chaperones [23].
	Allows inhibition of caspase 3 and regulation of the NLRP3 inflammasome [44].	Regulation of the NLRP3 inflammasome [41].	Decreased GABAergic activity from striatal neurons to GPi and SNpr [45].	Increased CD4+, CD8+, and CD45+ immune cells [46].	Allows inhibition of caspase 3 and regulation of the NLRP3 inflammasome [44].
	Increases fatty acid oxidation by increasing PUFA levels [40].	Beta-hydroxybutyrate activates PPARγ, thereby inhibiting nuclear NF-κβ expression. It also upregulates Nrf2 [47].	Increases activity of pro-apoptotic factors and prevention of inflammatory mediators such as interleukins and TNFα [21].	Decrease in pro-inflammatory cytokines such as IL-1β and IL-6, and increase in anti-inflammatory cytokines such as TGF-β [27].	Changes in gut microbiota [36].
	Changes in gut microbiota [48].	Regulation of genes that cause the production of excessive amounts of Aβ, such as *APP*, *PSEN1*, and *PSEN2* [49].	Changes in gut microbiota [50].	Changes in gut microbiota [35].	

## Data Availability

All clinical and statistical data and materials are available for the benefit of science.
